# Etomoxir regulates the differentiation of male germ cells by specifically reducing H3K27ac level

**DOI:** 10.1186/s12861-020-00237-x

**Published:** 2021-02-01

**Authors:** Yushan Xu, Jue Xie

**Affiliations:** Department of Blood Transfusion, The First Affiliated Hospital, College of Medicine, Zhejiang University, Hangzhou, 310003 China

**Keywords:** Gonocytes, Primordial germ cells, Proliferation, Differentiation, H3K27ac, CPT1A, Etomoxir

## Abstract

**Background:**

Fatty acid oxidation plays an important role in a variety of developing and mature organ systems. However, the role of this metabolic pathway in different stages of testis development remains unknown. Here, we elucidate the mechanisms by which fatty acid oxidation regulates the maintenance and differentiation of gonocytes and spermatogonial stem cells.

**Results:**

During E13.5-E15.5, male germ cells gradually enter the mitotic arrest phase, while the expression of CPT1A, a rate-limiting enzyme for fatty acid oxidation, gradually increases. Therefore, we treated pregnant mice (E13.5 to E15.5) with etomoxir, which is an inhibitor of CPT1A. Etomoxir-treated mice showed no difference in embryonic morphology; however, etomoxir-treated male gonocytes exited mitotic arrest, and cells of the gonad underwent apoptosis. In addition, etomoxir-treated mice at P7 displayed impaired homing of spermatogonia and increased cell apoptosis. We further demonstrated that inhibition of fatty acid oxidation in gonads was associated with gonocyte differentiation events and the histone modification H3K27ac.

**Conclusions:**

Inhibiting fatty acid oxidation can specifically reduce the level of H3K27ac in the reproductive crest, which may be the cause of the down-regulation of male differentiation-specific gene expression, which ultimately leads to the male primordial germ cells exited from mitotic arrest. Our work uncovers metabolic reprogramming during male gonadal development, revealing that it plays an important role in the maintenance of gonocytes in a differentiated and quiescent state during foetal testis development.

## Background

In mice, the germ line originates from primordial germ cells (PGCs) [1]; PGC precursors arise from the proximal epiblast at embryonic day (E) 6.25 and acquire the competence to become PGCs while actively repressed the somatic program [2]. Through the hindgut and body wall, PGC finally reaches genital ridge (GR) between E10.5-E11.5, XX and XY germ cells have undergone several rounds of proliferation and division [3]. By E13.5, Retinoic acid (RA) present in the female gonads induces the germ cells to enter the prophase of the first meiotic division.

By contrast, embryonic testes do not enter meiosis, and germ cells gradually slow down the rate of proliferation to produce mitotically inactive G0/G1 arrested gonocytes. PGCs colonization of gonads is critical for staging the arrest of meiosis or mitosis. The differentiation fate of germ cells must be consistent with the gender of gonads, which may otherwise lead to infertility and/or cancer [4]. The transition from mitosis to meiosis plays an important role in the development of germ cells and is strictly controlled, and disorder of this transformation is believed to play a role in germ cell tumour development [5]. Between E12.5 and E14.5, PGCs in the female embryonic gonad enter meiosis following signaling by retinoic acid. By contrast, the commitment to spermatogenesis of male PGCs involves inhibition of meiotic initiation, mitotic arrest and suppression of pluripotency. According to previous studies, ovarian or testicular somatic signaling mediates commitment. The switch between male fate in germ cells depends on the following signals: Cyp26b1 (cytochrome P450, family 26, subfamily b, polypeptide 1), Nanos2 (Nanos C2HC-type zinc finger 2), and Fgf9 (fibroblast growth factor 9). Cyp26b1 is a meiosis inhibiting factor, and male germ cells are protected from the RA secreted by the mesonephros by the RA-metabolizing enzyme Cyp26b1 [6]. It has been reported that in the testis of Cyp26b1 knockout mice, germ cells abnormally enter meiosis at E13.5 [7]. Nanos2 is expressed after the loss of Cyp26b1, and it plays an important role in promoting male primordial germ cell differentiation. Gonocytes of Nanos2 KO mice undergo normal mitotic arrest but re-enter mitosis and undergo apoptosis at E15.5 [8]; further, they are unable to correctly express male differentiation genes including stimulated by retinoic acid gene 8 (Stra8), synaptonemal complex protein 3 (Sycp3), and dosage suppressor of Mck1 homolog (Dmc1), at which point they begin to enter meiosis. Fgf9 inhibits male gonocyte entry into meiosis by downregulating Stra8, acting in a complementary manner to that of the Cyp26b1/RA pathway [9], while FGF9 has a sex-specific effect on germ cell survival: Fgf9 knockout mice cause gender reversal and induce male germ cell death at E12.5 [10].

Metabolism is the basic feature of life activities. Cellular metabolism provides an energy and material basis for physiological activities such as cell proliferation and differentiation [11–13]. The properties of chemical fluxes that change over a short period of time can modulate differentiation events through transcriptional levels and epigenetic properties. A large amount of data confirmed that there are highly dynamic metabolic changes during the embryonic development of mice, which affect gene expression through epigenetic modification and thus regulate proliferation and differentiation [11, 12, 14]. It is generally understood that the development of PGCs is facilitated by the expression of germ cell-specific transcription factors and characteristic epigenetic changes. However, the relationship between PGC differentiation and metabolic changes has rarely been studied.

It has been reported that aerobic oxidation of fatty acids can alter cell fate through unique molecular pathways. The level of fatty acid oxidation (FAO) regulates acetyl-CoA levels, controls key protein acetylation, and plays an important role in vascular and lymphatic endothelial cell fate control [11, 12]. Fatty acid oxidation is an important regulator of memory T cell fate, whereas effector T cell differentiation is more dependent on glycolysis [15]. In foetal gonads cultured in vitro, fatty acid oxidation plays an important role in the proliferation of female gonocytes through cell cycle regulation dependent on p53 [14]. However, there is a lack of similar data for male germ cells during the differentiation phase.

It has been reported that E13.5 germ cells have enriched levels of H3K27ac (histone H3 lysine 27 acetylation) and H3K4me3 (histone H3 lysine 4 trimethylation) at genes involved in sex determination, cell proliferation and differentiation, indicating that male differentiation is closely related to H3K27ac and H3K4me3 active histone methylation marks [16], and metabolism can regulate gene expression by regulating these histone modifications. Therefore, we make the hypothesis that this metabolic pathway in mouse testis may play an important role in regulating the maintenance and differentiation of gonocytes. In the present study, we examined the metabolic changes during the transition of PGCs to gonocytes. Carnitine acyltransferase I A (CPT1A), a mitochondrial transmembrane enzyme enabling fatty acid entry into the mitochondria, controls the rate-limiting step for the entire pathway. CPT1A was selected as the targeted protein of FAO. We demonstrated that Cpt1A levels increase during cell cycle arrest from E13.5-E15.5. Treatment with an inhibitor (etomoxir) showed that inhibiting FAO could make male germ cells exit mitotic arrest and impair development of spermatogonia. Inhibition of fatty acid oxidation leads to a decrease in the level of H3K27ac, which is associated with increased activation of transcription and is therefore defined as an active enhancer mark; the loss of this mark may be related to the decrease in acetylation of the male differentiation-specific genomic protein H3K27.

Thus, our work uncovers metabolic reprogramming during gonadal development and reveals that it plays an important role in the maintenance of gonocytes in a differentiated and quiescent state during foetal testis development.

## Result

### Male primordial germ cell differentiation and metabolic changes

**Fig. 1:**
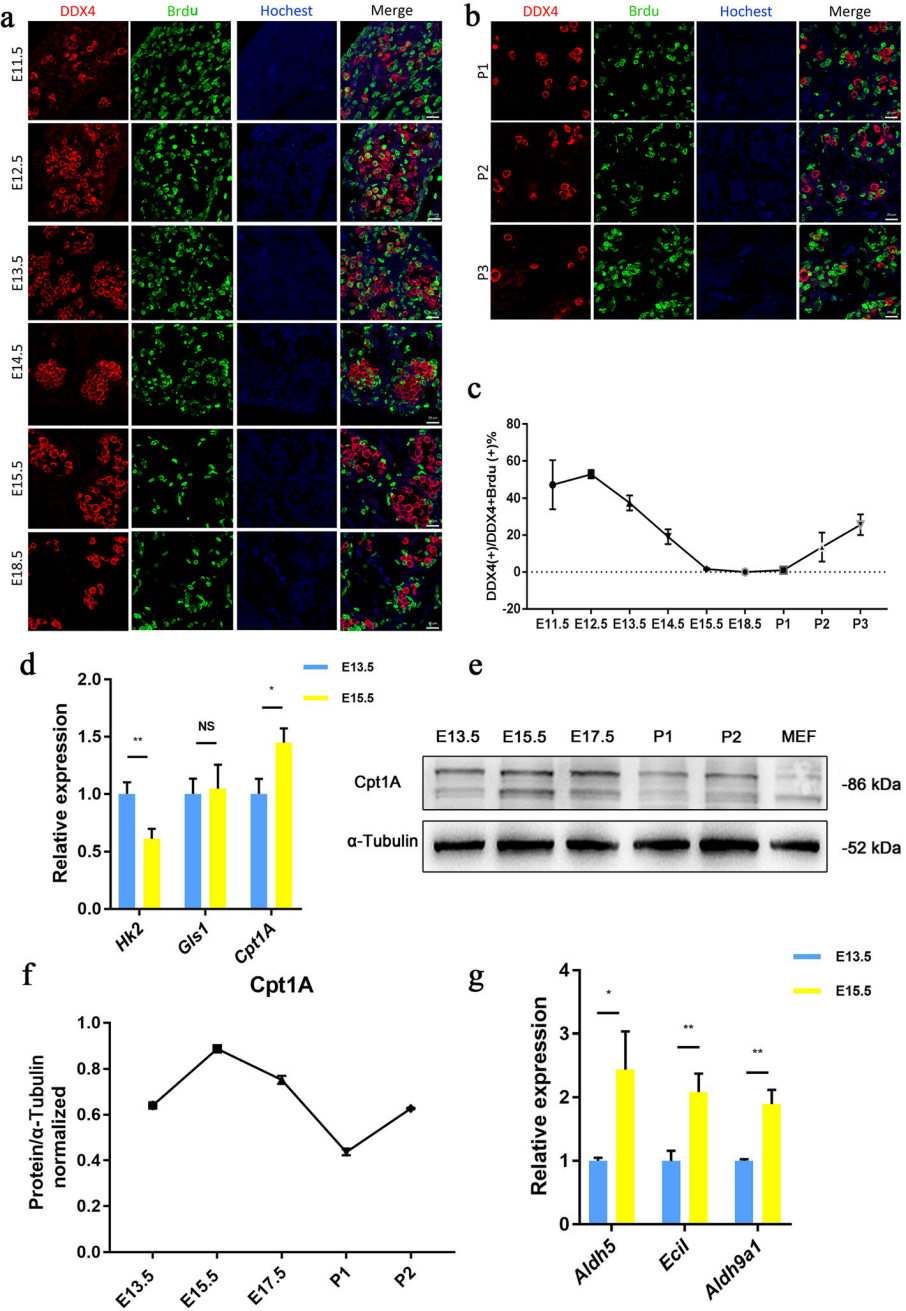
Male germ cells entering mitotic arrest are characterized by increased expression of fatty acid oxidation-related genes. **a**, **b** The period of mitotic arrest in male germ cells was determined. a: Incorporated BrdU (green), DDX4 (red) and Hoechst (blue) staining show the proliferation of male germ cells during the embryonic stage from 11.5 days (E11.5) to the third day after birth. Bar = 20 μm. **c** The germ cell proliferation ratio was calculated and the BrdU (+) DDX4 (+) number and DDX4 (+) ratio were determined at different time points. **d** Changes in the transcription levels of the genes of representative rate-limiting enzymes in three major metabolic pathways during E13.5-E15.5, as determined by RT-qPCR. Data normalized to Actin. **e-f** Dynamic changes in the protein levels of Cpt1a from E13.5-p2. (Note: The gels and blots were cropped.) **g** Changes in the transcription levels of fatty acid oxidation-related genes. Data normalized to 18 s. Data show mean ± SD and are representative of three independent experiments. (**p < 0.01, ***p < 0.001, ****p < 0.0001; ns, no significance; two-tailed unpaired Student’s t-test)

To better understand the cell cycle dynamics and the process of mitotic arrest in foetal male primordial germ cells, we used 5-bromo-2-deoxyuridine (BrdU) incorporation to analyze the DNA synthesis process, inferring the specific time at which mitotic arrest occurs. We analysed E11.5-P3 developing embryos to define the timing and dynamics of germ cell cycle arrest. Germ cells were identified with an antibody marker specific for DDX4 (DEAD-box helicase 4), a protein that is expressed specifically in developing mouse germ cells starting from E11.5. Using this analysis, we observed a high level of BrdU incorporation in E12.5 (53.0%) germ cells, demonstrating that at this stage, germ cells were rapidly cycling. In contrast to the high incorporation observed at E12.5, by E13.5, a significant proportion of the male germ cells had ceased to incorporate BrdU (37.4% were positive). By E15.5, the germ cells no longer incorporated BrdU (only 1.6% were positive), strongly indicating that by E15.5, male germ cells had entered mitotic arrest. We observed that BrdU incorporation restarted on P2 (13.5%), indicating that male germ cells had entered mitotic arrest recovery on P2 (Fig. 1a, b). We therefore propose that male germ cell differentiation begins at E13.5.

Metabolism is known to influence cell differentiation by regulating transcription and epigenetic mechanisms. Therefore, we hypothesized that metabolic reprogramming plays an important role in the differentiation of male primordial germ cells. We measured rate-limiting enzymes in three major metabolic pathways. Reverse transcription-qPCR (RT-qPCR) showed that *Cpt1a* (a rate-limiting enzyme for fatty acid oxidation) was higher at E15.5 than E13.5, and there was no difference between the expression of *Gls1* (a rate-limiting enzyme for glutamine metabolism) at the two time points; however, at E15.5, the expression of *Hk2* (a rate-limiting enzyme for glycolysis) was lower than it was at E13.5 (Fig. 1d). From E13.5-p2, the protein level of Cpt1a experienced a trend of increasing, decreasing, and increasing again (Fig. 1e, f), which was similar to the male germ cell mitosis arrest time trend (Fig. 1c). Therefore, we predicted that FAO plays an important role in the differentiation of male primordial germ cells. Next, using RT-qPCR we verified the expression trend of three enzymes of FAO from E13.5 to E15.5: aldehyde dehydrogenase family 5 (Aldh5), enoyl-coenzyme A delta isomerase 1 (Eci1), aldehyde dehydrogenase 9, subfamily A1 (Aldh9a1). The results showed that the transcriptional expression of FAO genes was upregulated at E15.5 compared with that of E13.5 (Fig. 1g).

### Etomoxir-treated male gonocytes display exit from mitotic arrest

**Fig. 2:**
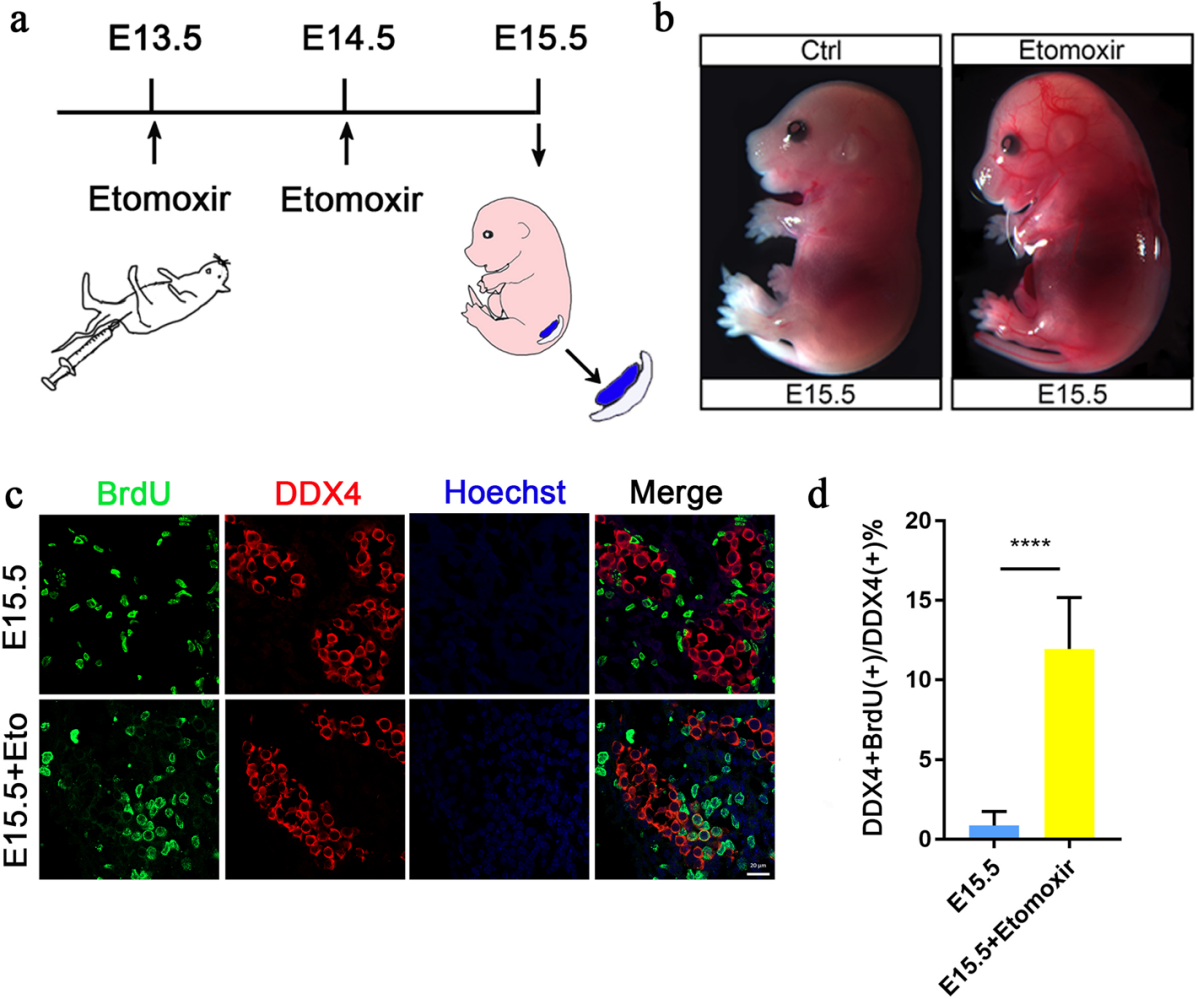
Fatty acid oxidation inhibition in a mouse model showing abnormal male germ cell differentiation. **a** Strategies for the use of pharmacological CPT1 receptor blockers (etomoxir). **b** Stereomicrographs at E14.5. **c**, **d** Confocal images of gonads at E15.5. Etomoxir-treated male gonocytes show increased incorporation of BrdU. (d are based on tests of ten embryos (samples) from each of the control and experimental groups. Bar=20 μm, **p < 0.01, ***p < 0.001, ****p < 0.0001; ns, no significance; two-tailed unpaired Student’s t-test)

To explore the effects of FAO on the differentiation of male PGCs and achieve rapid inhibition of FAO during the period of male PGC differentiation, we treated pregnant mice from E13.5 to E15.5 with the pharmacological CPT1 blocker etomoxir (we used a dose that, according to previous studies, effectively inhibited CPT1A activity without affecting embryonic blood vessels [12]) (Fig. 2a). Two mice per group, the experiment was repeated more than three times. Under a stereoscopic microscope, the Etomoxir-treated embryos showed no obvious developmental difference from the control embryos (Fig. 2b). Through the quantitation of BrdU and DDX4 double-positive germ cells in foetal male gonads at E15.5, we found that BrdU incorporation was elevated in the etomoxir-treated embryos at E15.5 (10.1%), whereas almost no BrdU incorporation was found in controls at E15.5 (Fig. 2c, d). These results indicate that inhibition of fatty acid oxidation causes male gonocytes to exit mitotic arrest.

### Etomoxir treatment impairs the homing of spermatogonia and increases cell apoptosis

**Fig. 3:**
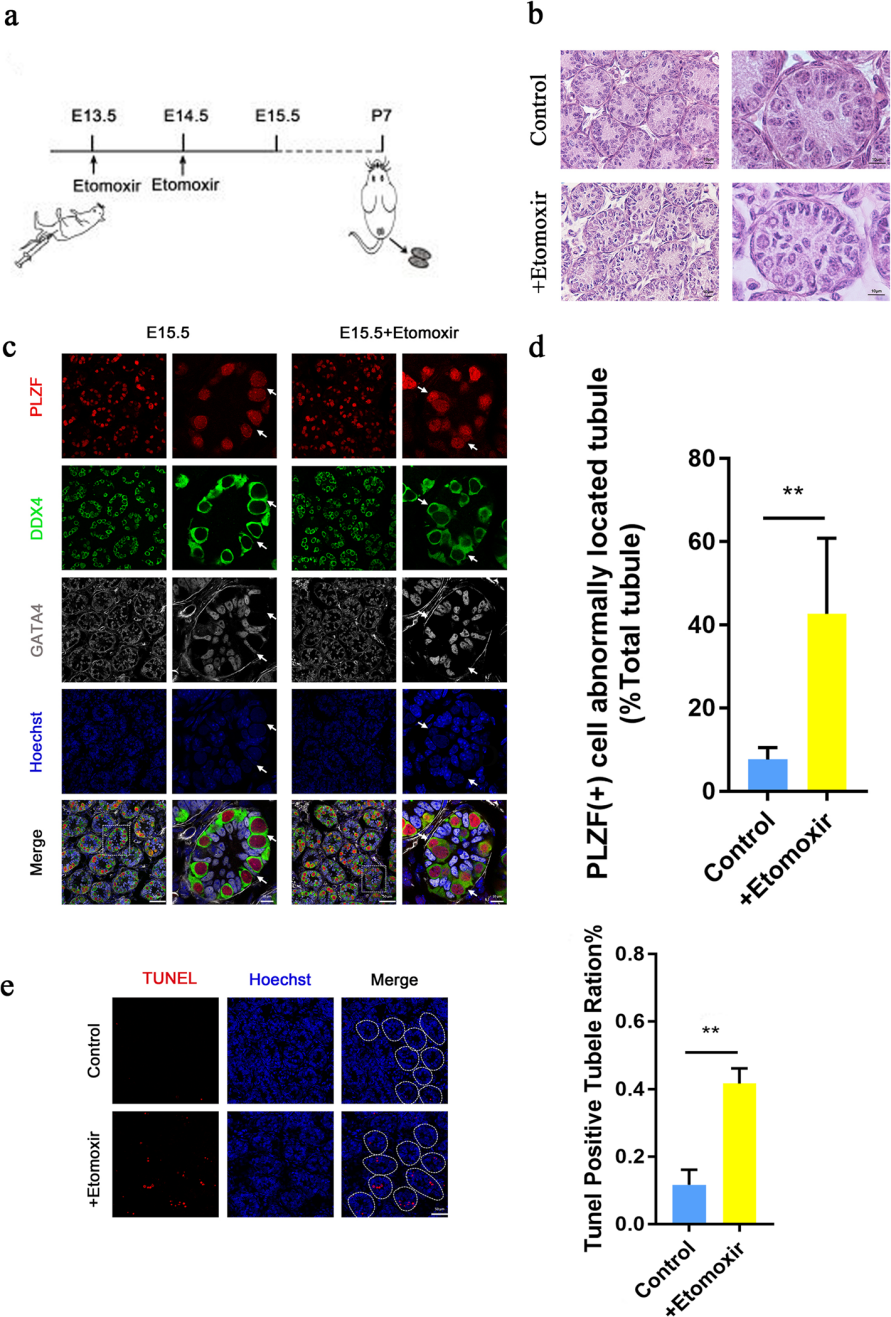
Etomoxir treatment impairs spermatogonia homing and increases germ cell apoptosis. **a** Strategies for the use of pharmacological CPT1 receptor blockers (etomoxir). **b** Images of sections of mouse testis stained using the H&E method at postnatal day 7. Bar=10 μm. **c** Confocal images of mice testes at postnatal day 7. The PLZF (+) cells of the control group (left) are localized to the substrate membrane (white arrow), while the PLZF (+) cells of the etomoxir-treated group (right) are dispersed in the lumen (white arrow). Bar = 20 μm. **d** Quantification of tubules with spermatogenesis (**p < 0.01). **e** Fluorescence TUNEL assay of the testes of the control and etomoxir-treated groups at P7. The apoptosis rate was calculated as the percentage of TUNEL-positive tubes. (d and e are based on tests of three embryos (samples) from each of the control and experimental groups. **p < 0.01, ***p < 0.001, ****p < 0.0001; ns, no significance; two-tailed unpaired Student’s t-test)

To explore whether the transitory inhibition of fatty acid oxidation during differentiation of male germ cells affected spermatogenesis, we intraperitoneally injected etomoxir from E13.5 for two consecutive days and stopped the injection at E15.5. Embryo development occurred on the 7th day after birth, and the testes of the postnatal mice were removed for observation (Fig. 3a). Through haematoxylin-eosin (HE) staining, the cells of the etomoxir-treated groups showed certain definite apoptotic features, including cell shrinkage, condensation and deep eosinophilia of the cytoplasm and pyknosis (Fig. 3b). At this time, the testicular lumen contains only spermatogonia and Sertoli cells. Germ cells were labelled with DDX4, and gonadal somatic cells were labelled with GATA binding protein 4 (GATA4). Undifferentiated spermatogonia were labelled with zinc finger and BTB domain containing 16 (PLZF). Immunofluorescence staining showed that the germ cells within control testes had migrated to the basement membrane at P7. In contrast, the germ cells of etomoxir-treated testes remained at the luminal side of the testicular tubules, indicating that they had lost the ability to locate the niche (Fig. 3c, d). Spermatogonia that are not located at the basement membrane, are known to enter apoptosis and eventually are engulfed by Sertoli cells. As evaluated by TUNEL assay, there were significantly more TUNEL-positive cells in the testicular tubules of the etomoxir-treated testis, as there were only a few TUNEL-positive cells in the testicular tubules of the control testis (Fig. 3e, f).

### Etomoxir-treated gonads reduced levels of histone H3K27 acetylation of the male differentiation-specific genomic protein

**Fig. 4:**
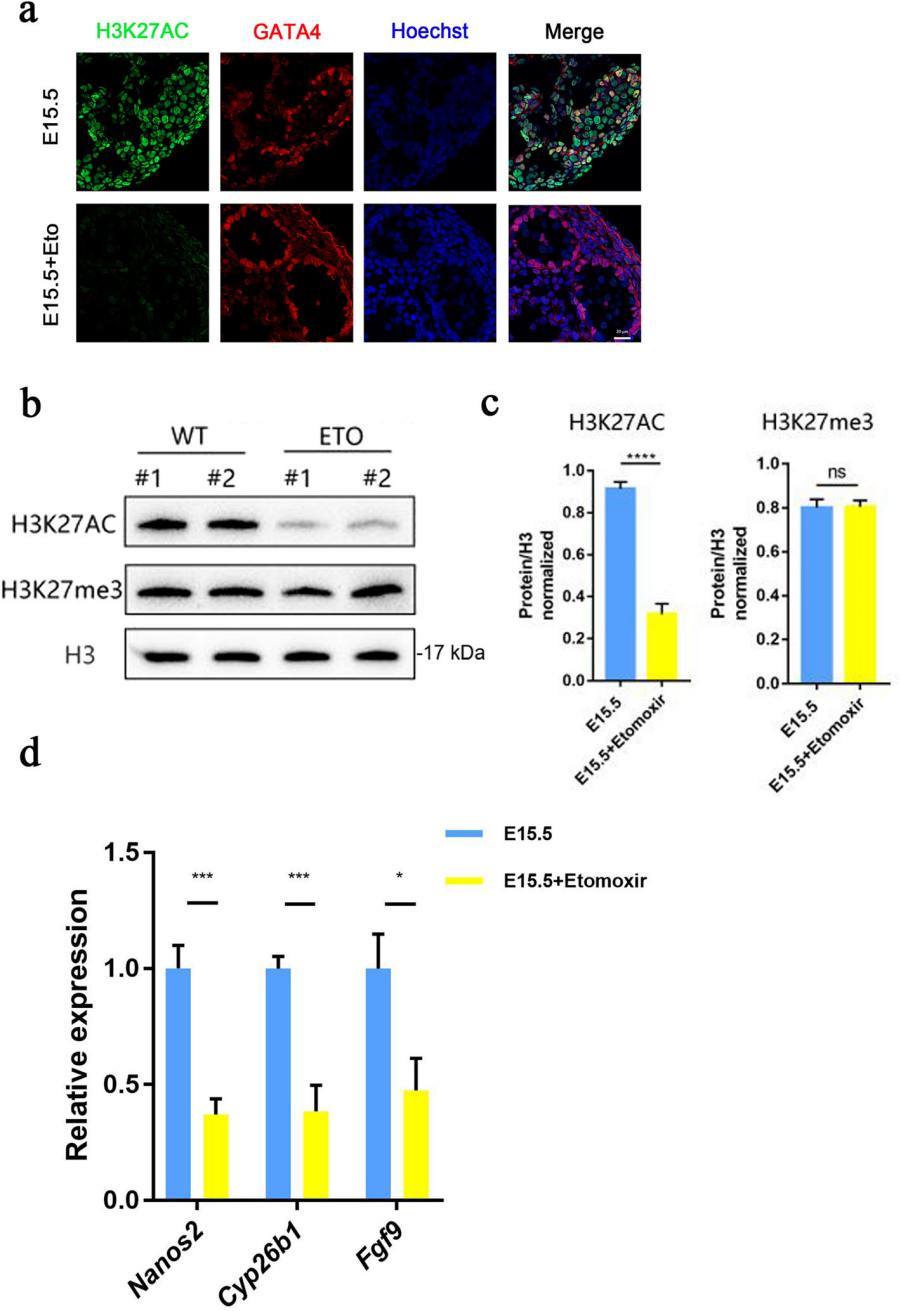
Etomoxir treatment reduces the levels of acetylation of the male differentiation-specific genomic protein H3K27, leading to downregulation of male differentiation-specific gene expression. **a**-**c** Immunofluorescence staining and western blot analysis were performed to examine the expression of H3K27ac and H3k27me3 in the control group and the etomoxir-treated group. (Note: The gels and blots were cropped.) **d** Changes in the transcription levels of male differentiation-specific genes between the control group and the etomoxir-treated group, as determined by RT-qPCR. Data normalized to 18 s. Greater than nine embryos (samples) from each of the control and experimental groups were tested to obtain the data in d. Perform three independent experiments on the data in d, e. (Bar = 20 μm, **p < 0.01, ***p < 0.001, ****p < 0.0001; ns, no significance; two-tailed unpaired Student’s t-test)

Fatty acid oxidation regulates specific protein acetylation by regulating acetyl-CoA levels, which may be one of the basic mechanisms by which fatty acid oxidation regulates cell and tissue development and differentiation [12, 17]. Furthermore, E13.5 germ cells have higher levels of H3K27ac and H3K4me3 at genes enriched for sex determination, cell proliferation and differentiation, indicating that the male differentiation is closely related to H3K27ac and H3K4me3 active histone methylation marks [16]. Analysis at E15.5 revealed that etomoxir reduced the level of H3K27ac (Fig. 4a,b,c). In summary, the inhibition of fatty acid oxidation reduced H3K27 acetylation in the male gonads.

**Fig. 5:**
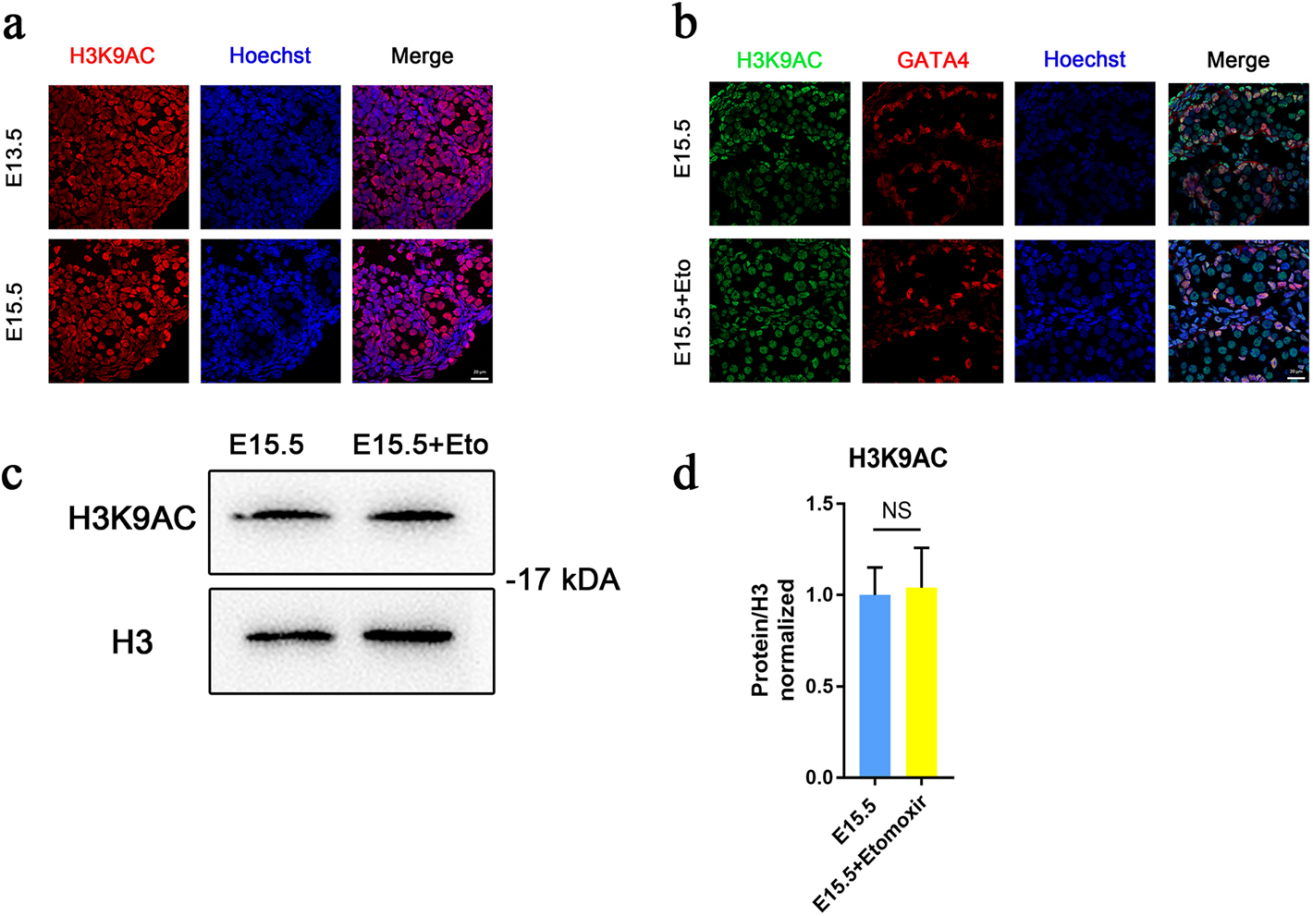
No significant change in the H3K9ac levels wasare observed when comparing control gonads and etomoxir-treated gonads from E13.5 to E15.5. **a** Immunofluorescence staining of gonads at E13.5, and E15.5 gonads, show shows the levels of H3K9ac. **b**-**d** Immunofluorescence staining and western blot analysis were performed to examine the expression of the H3K9ac between in the control groups and the etomoxir-treated groups. Perform three independent experiments on the data in D. Mention: The Gels and blots were cropped. (Bar = 20 μm, **p < 0.01, ***p < 0.001, ****p < 0.0001; ns, no significance; two-tailed unpaired Student’s t-test)

The decision to commit to a male germ cell fate depends on the environmental signals and expression of male differentiation genes. Specifically, Cyp26b1 produced by Sertoli cells and Leydig cells degrades retinoic acid, preventing expression of Stra8 and inhibiting meiosis. Nanos2, a factor blocking entry into meiosis and promoting male germ cell development, follows the expression of Cyp26b1. Fgf9 was shown to inhibit meiotic entry by downregulating the expression of Stra8. The Fgf9 and Cyp26b1 pathways act in a complementary way to regulate the level of Stra8 expression. In addition to RA, important factors in male differentiation mainly include deleted in azoospermia-like (Dazl), which has been proven to play an important role in sexual differentiation, and can inhibit pluripotency of PGCs, differentiation and apoptosis of somatic cells [18]. We performed RT-qPCR to detect the expression level of meiotic inhibition genes and found a significant decrease in the expression of Nanos2, Cyp26b1 and Fgf9 (Fig. 4d). So is the change in histone acetylation specific after metabolic changes? Our immunofluorescence results showed no change in H3K9ac levels from E13.5 to E15.5, and after treatment with Etomoxir (Fig. 5). Taken together, these results provide evidence that inhibition of fatty acid oxidation specifically reduced the level of H3K27ac, which may be the reason for the downregulation of male differentiation-specific gene expression.

## Discussion

Our data show that male PGCs begin to enter mitotic arrest at E13.5 and simultaneously initiate differentiation, during which time PGCs undergo changes in cell proliferation and differentiation. Regulatory genes, transcription factors and signal pathways form a complex network during PGC differentiation, and the role of metabolic changes in PGC differentiation has rarely been reported. By detecting the expression of key enzymes in the metabolic pathway, we found that lipid metabolism gradually increased during development from E13.5 (PGC stage) to E15.5 days (gonocyte stage), indicating that metabolic reprogramming occurred during PGCs differentiation. This suggests that when the proliferation and differentiation state changes, the utilization of metabolites by cells in the gonads also changes. To determine the relationship between metabolic reprogramming and differentiation events, we constructed a mouse model of fatty acid oxidation inhibition by intraperitoneal injection of etomoxir, an inhibitor of fatty acid oxidation, from E13.5-E15.5. Phenotypic analysis revealed that etomoxir-treated PGCs showed exit from mitotic arrest at E15.5 and increased apoptosis of cells in the gonad; disrupted spermatogonial homing was detected at P7, and increased cell apoptosis

**Fig. 6:**
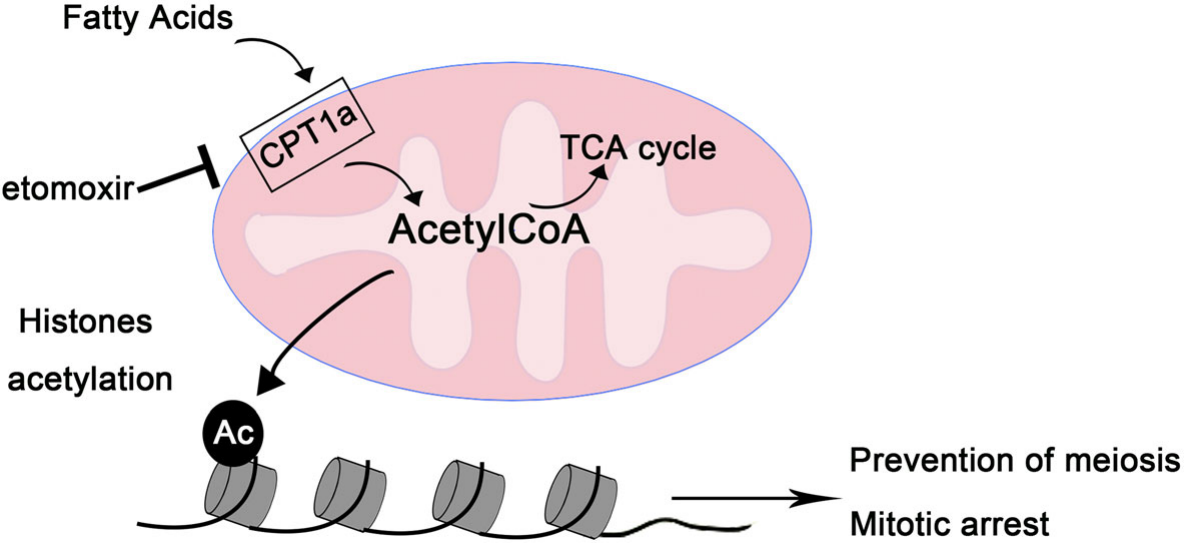
Etomoxir treatment leads to down regulation downregulation of male differentiation-specific gene expression. Changes in the utilization of metabolites by cells during male PGC differentiation, and upregulated increased fatty acid oxidation regulatesregulate male PGC differentiation-related genes by controlling the level of H3K27aC. In this way, these genes are activated in specific cells at specific timetimes to regulate the differentiation of PGCs

Male germ cell fate determination and differentiation are dependent on environmental signals and gene expression. In the process of male PGCs differentiation, maintaining mitotic arrest, preventing germ cells from entering meiosis and promoting the expression of male-specific differentiation genes are three events that affect each other. Evidence is starting to emerge that germline gene activation, including activation of meiotic genes, appears to be a common feature in a range of human neoplastic conditions [19]. Exit from mitotic arrest while activating meiosis-specific genes might be taken to imply meiotically primed cells are attempting a programmed entry into meiosis, albeit a flawed one [20]. There were similar phenotypes between etomoxir-treated mice and male differentiation-specific gene knockout mice. We verified the transcriptional repression of the male differentiation-specific genes Nanos2, Cyp26b1 and Fgf9 in the etomoxir-treated groups. Therefore, we suggest that at the stage of E13.5-E15.5, the inhibition of fatty acid oxidation lead to a decrease in the expression level of male differentiation-specific genes, which leads to abnormal exit from mitotic arrest. Whether abnormal meiosis is then initiated is what we will continue to study next. It has been reported that germline H3K9me2 is decreased, and H3K27me3 is upregulated before E13.5. The removal of H3K27me3 plays a critical role in the transcriptional initiation of developmental genes [21]. The results showed that H3K27ac levels gradually increased during E13.5-E15.5 in male gonads, suggesting that H3K27me3 is gradually shifted to H3K27ac in this process. Metabolism is known to regulate gene expression by regulating histone modifications in the following ways Increased CPT1A expression enhances the production of FAO-derived acetyl-CoA, for histone acetylation at lymphatic genes, an epigenetic process that enhances these sites by converting condensed chromatin into a more relaxed structure, regulating lymphatic proliferation and differentiation. In conclusion, our most notable finding is that FAO regulates male PGC differentiation genes by controlling the level of H3K27ac. In this way, these genes are activated in specific cells at specific times to regulate the differentiation of PGCs (Fig. 6).

Studies have shown that there is an off-target effect of etomoxir in excess of physiological concentrations [22, 23]; for example, an excess of etomoxir blocking macrophage polarization was traced to a depletion of free intracellular CoA, likely resulting from conversion of the pro-drug etomoxir into active etomoxiryl-CoA.

The polypharmacology of etomoxir is well-documented [24], and several promiscuous effects of the drug on metabolism have been identified in the literature, including inhibition of fatty acid and cholesterol synthesis, off-target transcriptional agonism, and depletion of cytoplasmic CoA [25]. Therefore, it is necessary to conduct a metabolomics screen of cells under different etomoxir concentrations. Therefore, the next focus is to study whether the concentration of etomoxir used in this experiment has an off-target effect and whether the phenotype exhibited by etomoxir treatment is caused by the inhibition of oxidation of fatty acids. The process of spermatogenesis, and consequently male fertility, are dependent upon testicular somatic cells. Leydig cells are essential for androgen production, whereas Sertoli cells, the main component of the so-called blood-testis barrier, are essential for the provision of an adequate and protected environment for germ cell development. The majority of germ cells are situated beyond the blood-testis barrier and rely on Sertoli cell production of factors to fuel their metabolism [26]. Sertoli cells have been shown to primarily utilize fatty acid oxidation rather than glucose to supply energy. Neonatal mice with a targeted disruption of Gata4 in Sertoli cells displayed an inability to establish and maintain the SSC pool, and apoptosis occurred in both gonocyte-derived differentiating spermatogonia [27]. We hypothesize that inhibition of fatty acid oxidation during embryonic development may affect the normal energy metabolism of Sertoli cells, leading to an increase in the apoptosis of Sertoli cells, which would cause an abnormal microenvironment in the testis after birth, affect the homing of spermatogonia, and ultimately leading to an increase in apoptosis of spermatogonia. It will be interesting to explore whether dietary modulation of fatty acids or supplementation with metabolites (acetate) can be used to promote spermatogenesis in pathological conditions.

## Conclusions

Sex determination is an integral part of reproduction. Inhibition of fatty acid oxidation reduced the level of H3K27ac, result in the downregulation of male differentiation-specific gene expression. So supplementation with metabolites can be a new targeting therapeutics to promote spermatogenesis in pathological conditions.

## Methods

### Mice and ethics statement

The C57BL/6 mice were obtained from the Animal Experimental Center of Nanjing Medical University. All animal work was approved by the Ethics Committee of Nanjing Medical University, China (IACUC-1601273). Mice were kept within a strictly controlled environment of 20–25C, 50–70% humidity, 12/12 h light/dark cycles, with free access to food and water. For timed matings to obtain fetal samples, male and female mice were paired together and checked for the presence of a vaginal plug. The day a vaginal plug was detected was considered E0.5. At the appropriate day of embryonic development after vaginal plug, the pregnant mice were euthanized by cervical dislocation, and the uteri were removed.

Randomly select the control group and the experimental group in the same litter of mice, for experiments assessing the role of etomoxir in mouse embryonic development at E15.5, 30 mg kg–1 etomoxir was injected intraperitoneall in healthy pregnant dams daily from E13.5–E15.5. Choose a dose that does not affect embryo development, while the control group was injected intraperitoneally with the same dose of water.

All experiments involved in the article chose to use the entire gonads (testis).

### Antibodies

Rabbit anti-Hk2 (CST, 2867S); Rabbit anti-DDX4/MVH (Abcam, ab13840); Rabbit anti-Cpt1A (); mouse anti-CPT1A (Proteintech, 66,039–1-Ig); rabbit anti- Stra8 (Abcam, ab49602); rabbit anti- Sycp3 (Abcam, ab15093); mouse anti- Gata4 (santa cruz, sc-25,310); rabbit anti-acetyl histone H3 lysine 9 (CST, 9649S); rabbit anti-acetyl histone H3 lysine 27 (Active Motif, 39,133); Rabbit anti-γ-H2AX (Abcam, ab11174); Rabbit anti- PLZF (Abcam, ab49602); Rat anti- BrdU (Abcam, ab6326); Appropriate AlexaFluor-594, − 488, − 555 or − 647 conjugated secondary antibodies were used (Thermo).

### Histology

In order to evaluate the histological characteristics of P7 testes, the testes were placed in MDF solution overnight at room temperature, rinsed several times in 70% ethanol the next day, and then dehydrated stepwise in graded ethanol, and finally embedded in paraffin with a section thickness of 5 μm, and staining with hematoxylin–eosin (HE) according to standard protocols. A minimum sample size =1 is required for a single experiment. Data are representative of three independent experiments.

### Immunostaining

The mice were euthanized by cervical dislocation. Immediately after removing the testes, they were fixed in 4% formaldehyde (PFA) and then dehydrated using a sucrose gradient, as previously described [27]. For immunofluorescence, sections were blocked with blocking buffer (donkey serum, 0.3% Triton X-100 in PBS) for 2 h, and incubated with primary antibody at 4 °C overnight. The sections were washed and incubated with a secondary antibody (Thermo) for 2 h, and then counterstained with Hoechst (Sigma,) to identify nuclei. A minimum sample size =1 is required for a single experiment. For each staining and time point analysis, at least three separate images from separate fetuses or pups are used.

### BrdU incorporation assay

BrdU labeling and detection were conducted, as previously described [12]. Briefly, mice were injected intraperitoneally with 100 mg/kg body weight of BrdU (Sigma, B5002) 2 h before sacrifce. The gonads or testes were then removed. Treat the gonad sections according to the procedure described previously, except that sections were denatured with 2 N HCl for 5 min before incubating with primary antibodies. The percentages of DDX4(+) PGCs positive for BrdU label were determined using an LSM800 confocal microscope (Zeiss). Data are representative of three independent experiments.

### TUNEL assay

A TUNEL assay was conducted using an TUNEL BrightRed Apoptosis Detection Kit (Vazyme, A113–03). Sections were counterstained with Hoechst to identify the nuclei. Data are representative of three independent experiments.

### Western blot

Protein extraction and immunoblot analysis was performed using a modified lysis buffer (1 M Tris-HCl (pH 7.5), 5 M NaCl, 0.5 M EDTA (pH 8.0), 10% NP 40, 12.5% Deoxychalate,20% SDS) in the presence of protease inhibitors (Sangon Biotech).^.^ The proteins were electrophoresed under reducing conditions in 10% or 12.5% SDS-PAGE gels and transferred to nitrocellulose membranes. The blots were blocked in 5% NON-Fat Powdered Milk (Sangon Biotech) and incubated overnight at 4 °C with the primary antibody, followed by incubation with the secondary antibody for 2 h at room temperature. Appropriate secondary antibodies were from Invitrogen. Signal was detected using the ECL system (fdbio) according to the manufacturer’s instructions. Unless otherwise indicated, α-Tubulin was used as loading control. Densitometric quantifications of bands were done with NIH Image J software. Data are representative of three independent experiments. A minimum sample size =3 is required for a single experiment.

### Histone extraction

Gonads were collected, grinding and resuspended in 500 μl cold hypotonic lysis buffer. Then rotated for at least 30 min at 4 °C. After centrifugation at 15,000 g for 10 min at 4 °C, the pellet was resuspended in 400 μl 0.2 M H_2_SO_4_, 4 °C overnight. Next day, after centrifugation at 15,000 g for 10 min at 4 °C, histones were precipitated by addition of 33% trichloroacetic acid for at least 30 min placed on ice, followed by centrifugation at 16,000 g for 10 min at 4 °C. The pellet was washed with acetone. Histone proteins were resuspended in ddH2O.

### Real-time quantitative PCR

The frozen gonads were crushed using a micro-homogenizer, and the isolation of total RNA using Trizol (Invitrogen) according to the manufacturer’s protocol. RNA samples were subjected to reverse transcription using a RT reagent Kit (Takara). The reactions were run in triplicate in three independent experiments. Samples CT values were normalized to the corresponding Actin/18 s CT values, and relative expression levels were calculated using the ΔΔCT method, The relative gene expression levels were compared with those in the controls. The primer sequences are provided in Table 1. Data are representative of three independent experiments. A minimum sample size =3 is required for a single experiment.

**Table 1:**
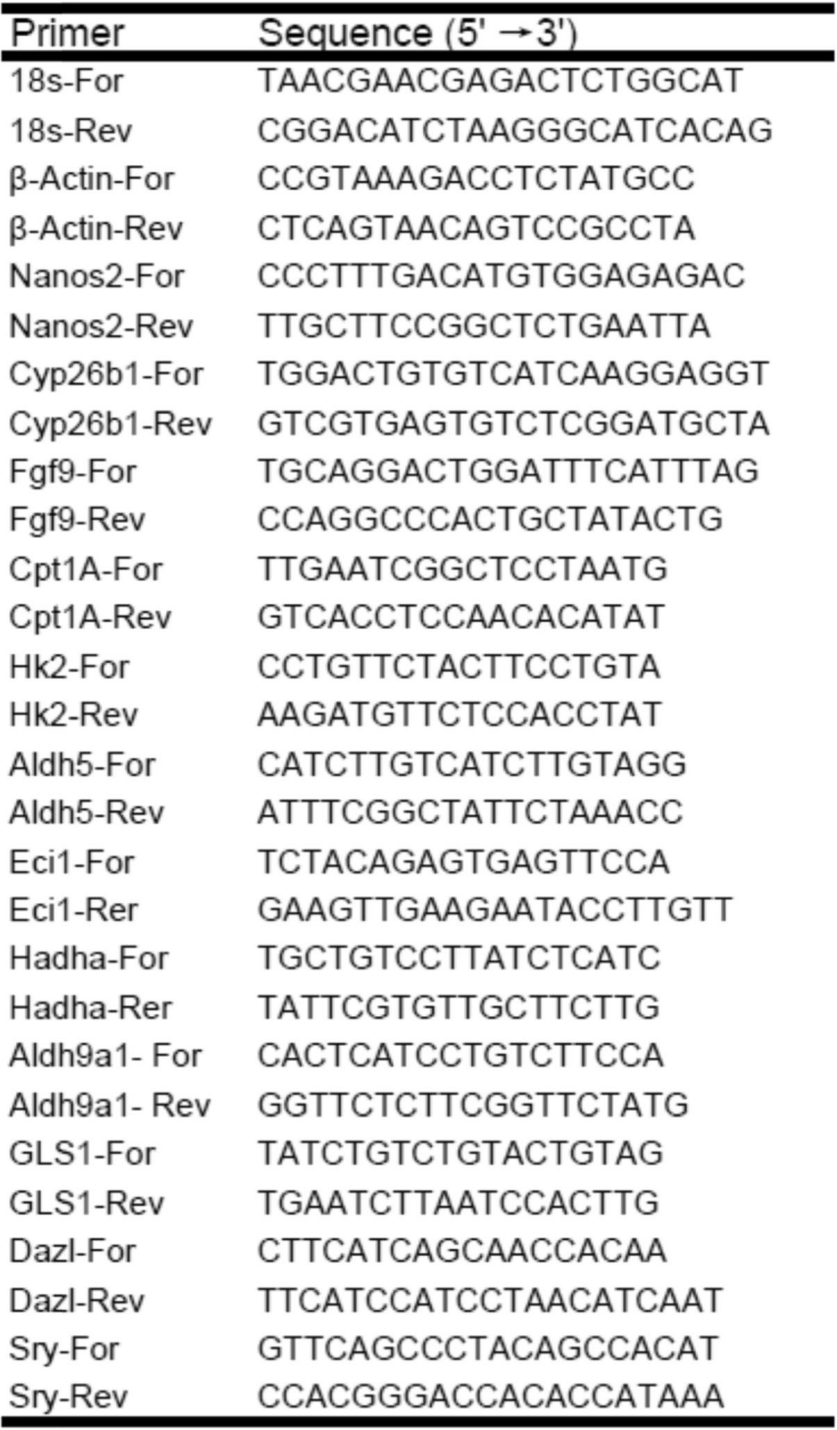
Primers sequences used in the RT-qPCR

### Statistics

For experiments involving a single pair of conditions, statistical significance between the two sets of data were analyzed with an unpaired-t-test (Mann-Whitney) with Prism5 (GraphPad software). For datasets containing more than two samples, one-way analysis of variance (ANOVA) was used. Sample sizes of sufficient power were chosen on the basis of similar published research. At least 3 samples for each experimental conditions were analyzed. Statistically significant differences are reported at *P < 0.05, **P < 0.01, ***P < 0.005, ****P < 0.001.

## Data Availability

The datasets generated and analysed during the current study are available in the Figshare repository, 10.6084 / m9.figshare.13347215.

## Abbreviations

PGCs: Primordial germ cells
E6.25: Embryonic day 6.25
GR: Genital ridge
RA: Retinoic acid
Cyp26b1: Cytochrome P450, family 26, subfamily b, polypeptide 1
Nanos2: Nanos C2HC-type zinc finger 2
Fgf9: Fibroblast growth factor 9
Stra8: Stimulated by retinoic acid gene 8
Sycp3: Synaptonemal complex protein 3
Dmc1: Dosage suppressor of Mck1 homolog
FAO: Fatty acid oxidation
H3K27ac: Histone H3 lysine 27 acetylation
H3K4me3: Histone H3 lysine 4 trimethylation
CPT1A: Carnitine acyltransferase I A
BrdU: 5-bromo-2-deoxyuridine
DDX4: DEAD-box helicase 4
Gls1: Glutaminase-1
Hk2: Hexokinase 2
Aldh5: Aldehyde dehydrogenase family 5
Eci1: Enoyl-coenzyme A delta isomerase 1
Aldh9a1: Aldehyde dehydrogenase 9, subfamily A1
Hadha: Hydroxyacyl-coenzyme A dehydrogenase/3-ketoacyl-coenzyme A thiolase/enoyl-coenzyme A hydratase, alpha subunit
HE: Haematoxylin-eosin
GATA4: GATA binding protein 4
TUNEL: Terminal dexynucleotidyl transferase (TdT)-mediated dUTP nick end labeling
Dazl: Deleted In Azoospermia Like
